# Gamete Gamble: Phthalate Alters Germ Cell Development

**Published:** 2009-01

**Authors:** Bob Weinhold

Di-2-ethylhexyl phthalate (DEHP), one of the most abundant phthalates produced, has been incorporated into flexible plastic products such as food containers and packaging, toys, medical equipment, and home and garden products. DEHP is being phased out of some products because of growing concern about its potential health effects. A French team has now established the first tangible link between one phthalate, the DEHP metabolite mono-2-ethylhexyl phthalate (MEHP), and altered human germ cell development **[*EHP* 117:32–37; Lambrot et al.]**.

The French team acquired testes from morphologically normal fetuses of women undergoing legal abortion during weeks 7 to 12 of gestation. Using an organotypic culture system, they exposed the testes for 3 days to one of three concentrations of MEHP: 10^−6^, 10^−5^, or 10^−4^ M. The highest concentration was 2 orders of magnitude higher than that known by the authors to occur in humans; the lowest was the same order of magnitude as that found in human milk in Finland, which reached 1,410 μg/L. Biomonitoring data for 2005 published by the Centers for Disease Control and Prevention (CDC) showed that MEHP in the urine of U.S. residents reached 52.1 μg/L (or 10^−8^ M).

At the highest concentration, the authors found that exposure reduced germ cell numbers by 40%. The sharp reduction occurred via an increase in apoptosis, or programmed cell death, without any effect on proliferation. The authors note that the plunge in numbers is crucial because the germ cells formed during fetal life—which will go on to become ova or sperm—help determine adult fertility.

The highest concentration of MEHP also significantly reduced the messenger RNA expression of anti-Müllerian hormone, which plays a key role in the development of certain cells into male reproductive organs, usually during week 8 of fetal development. The lowest concentration of MEHP tested didn’t show adverse effects for the pathways analyzed.

The general population is routinely exposed to many types of phthalates, with at least one metabolite, monoethyl phthalate, documented in urine by the CDC at concentrations of 10^−6^ M. The authors suggest that researchers should investigate additional phthalates and interactive effects, other concentrations and periods of exposure, different time periods of fetal development, and additional pathways. They also note that their findings conflict with some results from animal studies. For instance, there were no MEHP effects on testosterone production in this study, but testosterone suppression has occurred in rats exposed to phthalates. Such discrepancies may be due to differences between species, they say.

